# Molecular mechanisms of TGFβ-mediated EMT of retinal pigment epithelium in subretinal fibrosis of age-related macular degeneration

**DOI:** 10.3389/fopht.2022.1060087

**Published:** 2023-03-01

**Authors:** Fumiaki Higashijima, Mina Hasegawa, Takuya Yoshimoto, Yuka Kobayashi, Makiko Wakuta, Kazuhiro Kimura

**Affiliations:** Department of Ophthalmology, Yamaguchi University Graduate School of Medicine, Ube, Yamaguchi, Japan

**Keywords:** TGF-β, myofibroblast, epithelial-mesenchymal transformation, subretinal fibrosis, chronic inflammation

## Abstract

Age-related macular degeneration (AMD) is one of the leading causes of blindness in the elderly, affecting the macula of the retina and resulting in vision loss. There are two types of AMD, wet and dry, both of which cause visual impairment. Wet AMD is called neovascular AMD (nAMD) and is characterized by the formation of choroidal neovascular vessels (CNVs) in the macula. nAMD can be treated with intravitreal injections of vascular endothelial growth factor (VEGF) inhibitors, which help improve vision. However, approximately half the patients do not achieve satisfactory results. Subretinal fibrosis often develops late in nAMD, leading to irreversible photoreceptor degeneration and contributing to visual loss. Currently, no treatment exists for subretinal fibrosis, and the molecular mechanisms of fibrous tissue formation following neovascular lesions remain unclear. In this review, we describe the clinical features and molecular mechanisms of macular fibrosis secondary to nAMD. Myofibroblasts play an essential role in the development of fibrosis. This review summarizes the latest findings on the clinical features and cellular and molecular mechanisms of the pathogenesis of subretinal fibrosis in nAMD and discusses the potential therapeutic strategies to control subretinal fibrosis in the future.

## Introduction

Age-related macular degeneration (AMD) is a progressive degeneration of the macula in people aged 50 years and older and is the leading cause of blindness in the elderly in developed countries (1). Early stages of AMD are characterized by large drusen and retinal pigment epithelial (RPE) cell damage (2). AMD is classified into wet and dry types (3). Dry AMD is known as geographic atrophy of the macula, whereas wet AMD is characterized by the development of abnormal blood vessels underneath the macula and is called neovascular AMD (nAMD.) The first-line treatment for nAMD is intraocular injection of a vascular endothelial growth factor (VEGF) inhibitor (such as anti-VEGF). Although this treatment can prevent or improve vision loss, a certain proportion of recurrent or inoperable cases is also observed. More importantly, approximately half of the patients show progressive fibrosis and atrophy of the macula, which is not adequately treated (4). The pathogenesis of macular fibrosis in nAMD is not completely understood, and no effective treatment exists. This review describes the clinical manifestations and underlying mechanisms of macular fibrosis secondary to nAMD, focusing on the origin of myofibroblasts, pathways involved in their induction and activation, and regulatory mechanisms of factors working downstream.

## Subsections relevant for the subject

### Clinical features of subretinal fibrosis in nAMD

**Figure 1:**
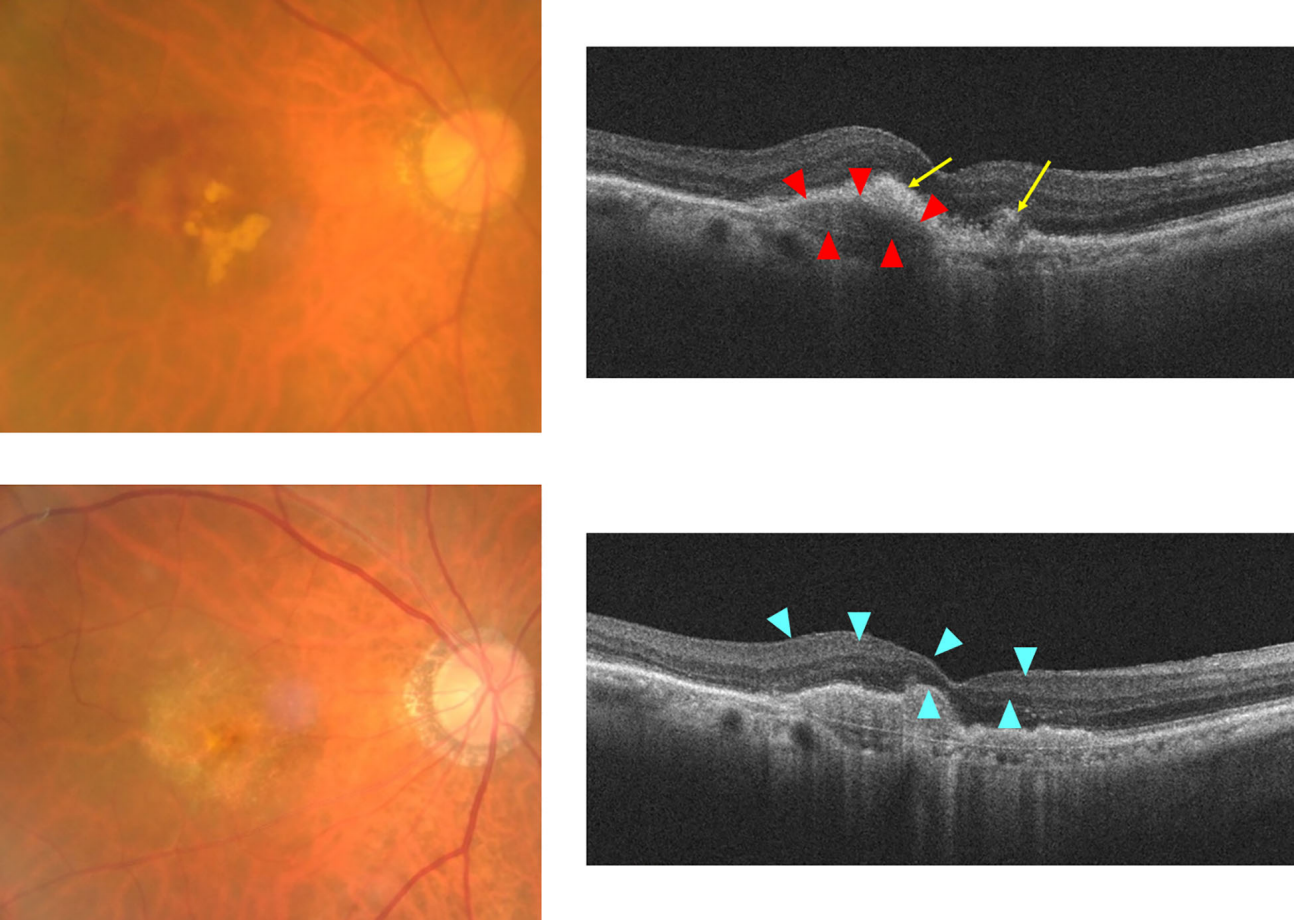
Formation of SHRM during anti-VEGF therapy for nAMD case. Upper: before treatment, Lower: 12 months after first treatment. Right: fundus color photo, Left: optical coherence tomography (OCT) image. Grayish-white neovascular vessels with small amounts of hemorrhage and white exudative lesions are observed in the macula of the left eye before anti-VEGF therapy. The OCT image shows RPE elevation due to fibrovascular membrane with choroidal neovascularization (red arrowheads), surrounding the hyperreflective lesion (yellow arrows) with an indistinct margin suggestive of hemorrhage and fibrin deposition. Twelve months after initial treatment, white fibrosis tissue and depigmentation associated with retinal atrophy were observed. OCT images show the formation of sharply marginated SHRM (blue arrowheads) above the Bruch’s membrane at the macula.

nAMD causes blindness due to the development of choroidal neovascularization (CNV). CNV causes exudation of fluid, hemorrhage, and fibrin deposition on the macula, forming subretinal fibrosis tissue at the end stages of nAMD (5). Patients with advanced nAMD often show subretinal hyperreflective material (SHRM) on optical coherence tomography (OCT), which can noninvasively take tomographic images of the retina. SHRM is a subretinal deposit comprising neovascular tissue, fibrosis, exudate, vitelliform material, hemorrhage, and reticular pseudodrusen and is reportedly associated with subretinal fibrosis (6, 7). Treatment options for nAMD include VEGF-targeted therapy, photodynamic therapy, and laser photocoagulation (3). Anti-VEGF therapy has been found to effectively prevent vision loss in patients with nAMD; however, sometimes, subretinal fibrosis occurs afterward (8). **Figure 1** shows SHRM formation and macular fibrosis during treatment with anti-VEGF therapy of a patient with nAMD. OCT showed CNV with hemorrhage and fibrin deposition before treatment, which were improved by anti-VEGF therapy; however, the CNV became SHRM, and visual acuity decreased after 1 year. The results of a CATT study of 1059 eyes reported that scarring was observed in 480 eyes (45.3%) during the 2-year treatment course and that SHRM is a risk factor for fibrosis (4) Subretinal fibrosis has a higher incidence in nAMD patients with active CNV and is thought to occur spontaneously with progression of nAMD with inflammation and exudative changes (9). On the other hand, it has also been suggested that anti-VEGF therapy may cause maturation of the neovascular complex, SHRM, which may transform into subretinal fibrosis (10). In a previous report before the introduction of anti-VEGF therapy, disciform scars were observed in 310 of 760 eyes (40.8%) of nAMD patients (11). Whether subretinal fibrosis is accelerated by anti-VEGF therapy or whether subretinal fibrosis progresses even with anti-VEGF therapy is an issue to be clarified by future studies. Subretinal fibrosis destroys retinal and choroidal structures and inhibits the transport of oxygen and nutrients from the choroid to photoreceptor cells, resulting in adverse effects on vision. The development of subretinal fibrosis is associated with multiple biological processes including various cell components such as infiltrating inflammatory cells, resident cells such as glial cells (Müller cells), retinal pigment epithelial (RPEs) cells, and retinal and choroidal vascular endothelial cells, choroidal fibroblasts and macrophages (12, 13). Subretinal fibrosis is a clinically significant problem; however, its molecular etiology remains unclear and there is no effective treatment.

### Pathophysiology of subretinal fibrosis in nAMD

When tissue is injured, the wound healing process is rapidly activated to maintain tissue homeostasis (14). In this process, inflammatory cells, endothelial cells, fibroblasts, and resident cells are mobilized at the site of injury and are transformed into myofibroblasts, which are then activated. Myofibroblasts have both the fibroblast-like morphological features found in tissues and smooth muscle cell-like contractile features. Myofibroblasts are characterized by expression of αSMA as a marker protein and excessive synthesis and secretion of extracellular matrix such as collagens (types I, III, IV, and V), glycoprotein (fibronectin, laminin, tenascin etc), proteoglycans (perlecan, decorin etc) and elastin (15–17). It is well known from previous studies that there is variability in the phenotype of fibroblasts and myofibroblasts in tissues and foci. Recently, by studying various cell populations at single cell resolution with single cell analysis, it is possible to identify new cell clusters with unique transcriptional signatures (18). Therefore, the advent of new single cell analysis has revealed the heterogeneity of fibroblast and myofibroblsts cell groups. Recent single-cell analysis studies have inferred new markers (including hedgehog interacting protein (Hhip), asporin (Aspn), and musculoskeletal embryonic nuclear protein 1 (Mustn1)) that are more abundantly expressed than αSMA in myofibroblasts, and have been reported to be superior to αSMA in distinguishing myofibroblasts (19). Furthermore, in pulmonary fibrosis, αSMA-positive cells increase dramatically during fibrosis, but the cells that comprise them are a mixture of matrix fibroblast subtypes as well as myofibroblast subtypes, and matrix fibroblasts are more numerous (19). These results suggest that among the αSMA-positive fibroblasts that have been considered myofibroblasts, there may be a mixture of pathogenic fibroblasts that are formed by the tissue- and disease-specific inflammatory environment. The nature and characteristics of fibroblasts and myofibroblasts are finally being defined not simply by a few markers, but by looking at the transcriptional program as a whole. Whether these clusters are truly distinct subpopulations or represent the same population but different states remain to be elucidated. Activated myofibroblasts remodel the injured area and promote structural and functional improvement of the tissue. Under physiological conditions, myofibroblasts disappear by apoptosis with wound healing (20). However, when the balance in this tissue repair process is disrupted by chronic inflammation or persistent injury, myofibroblasts remains continuously activated, causing excessive secretion and deposition of ECM proteins such as collagen and fibronectin, resulting in the formation of fibrous tissue (21).

Previous studies have shown that the formation of fibrotic proliferative tissue on or under the retina in nAMD involves persistent chronic inflammation and various cellular components, including infiltrating inflammatory cells (macrophages), glial cells (Müller cells, astrocyte and microglia), retinal pigment epithelial cells (RPE), and resident cells such as vascular endothelial cells (22, 23). RPE cells migrating into the pathological CNV tissue of nAMD patients express α-smooth muscle actin (α-SMA), a marker molecule for myofibroblasts (23). Under conditions of persistent chronic inflammation, RPE, inflammatory cells, endothelial cells, and glial cells are transformed into myofibroblasts, which are further activated (13, 24). The vascular component of the CNV lesion regresses, followed by an increase in the fibrous component, forming subretinal fibrosis (7, 25).

Thus, previous studies have shown that multiple cell types are involved in the formation of subretinal fibrosis in nAMD. Because myofibroblasts are not normally observed in the macula, it is reasonable to assume that all cases of subretinal fibrosis that occur secondarily in nAMD arise from myofibroblast progenitor cells (13, 26).

Epithelial-mesenchymal transition (EMT) causes epithelial cells to differentiate into mesenchymal cells, lose their epithelial cell characteristics and gain migratory or proliferative abilities, and exhibit an increased ECM synthesis capacity, thereby contributing to numerous phenomena such as development, wound healing, fibrosis, and cell migration (27, 28). EMT is characterized using the expression of α-SMA, collagen type I (COL1), and fibronectin, as well as the connective tissue growth factor (CTGF) (29, 30). RPE cells are transformed into myofibroblasts by epithelial-mesenchymal transition (EMT), which contributes to various retinal proliferative diseases including subretinal fibrosis (3). *In vivo* model of proliferative vitreoretinopathy induced by injecting RPE cells into the vitreous, RPE cell migration and EMT to fibrotic cells resulted in the formation of a proliferative membrane on the retinal surface. This supports the involvement of EMT of RPE in the formation of proliferative membranes in proliferative vitreoretinal diseases (31). EMT is also an important process including liver, lung and other organs fibrosis (32). Bloch et have reported that the development of subfoveal fibrosis in nAMD was mainly associated with classic CNV, in which the neovascular penetrates Bruch’s membrane-RPE complex, entering the subretinal space (33). Consequently, injured RPE cells become myofibroblasts by EMT process and may contribute to subretinal fibrosis. In addition, many myofibroblast-like stromal cells in surgically resected tissue fragments containing CNV are positive for both α-SMA and cytokeratin, and there was also a gradation of α-SMA and cytokeratin immunoreactivity from sites adjacent to the normal RPE cell layer to CNV (34). Moreover, RPE cells can undergo EMT under experimental conditions (35–37). In nAMD, RPE is converted to myofibroblasts, which may have a central role in subretinal fibrosis

### TGF-β-mediated signaling regulation in EMT of RPE

In subretinal fibrosis of nAMD, myofibroblasts cause pathological over-deposition of ECM proteins, which coupled with increased myofibroblast activity results in chronic inflammation as well as macrophage and immune-cell infiltration. In nAMD lesions, infiltrating macrophages and RPE cells are phenotypically altered to myofibroblasts, which are activated and become a major source of chemokines and growth factors (26, 38). These retinal microenvironments are important in the pathogenesis of subretinal fibrosis in nAMD.

Under these microenvironments, inflammatory cytokines such as TNF-α, IL-8, and IL-6 and growth factors such as TGF-β, platelet-derived growth factor (PDGF), CTGF, and sphingosine 1-phosphate (S1P) are secreted and contribute to further development of subretinal fibrosis (26). Among them, TGF-β acts as one of major effectors during fibrosis (39).

Factors such as transforming growth factor-β (TGF-β), CTGF, and thrombin are known to trigger EMT of RPE. Gamulescu et reported that TGFβ2 dose-dependently induced cultured human RPE to differentiate into α-SMA-positive myofibroblast-like cells (40). CTGF is downstream of TGFβ and promoted EMT of RPE in proliferative vitreoretinopathy (PVR) through the Phosphatidylinositol-3-kinase (PL3K/AKT) pathway (41). Thrombin promotes morphological changes and migration of RPE cells *via* phosphorylation of FAK and is involved in the formation of proliferative membranes (42). It has also been reported that EMT of RPE cells is activated *via* the TNFα/NF-κB/Snail pathway in a mouse model of proliferative vitreoretinopathy (43). EMT of RPE is induced through various signaling pathways. Among them, TGF-β acts as one of major effectors during fibrosis (39). In fact, clinical data have confirmed that TGFβ levels in the anterior chamber fluid and vitreous humor of nAMD patients are elevated (44, 45).

Transforming growth factor–beta (TGF-β), a multifunctional cytokine, is detected in the pathological tissue sections from nAMD patient and seems to be related to the process of subretinal fibrosis formation in nAMD (46). TGF-β also have involved in the induction of EMT in RPE, and up-regulates the expression of Matrix metalloproteinases (MMPs) - including MMP-2 and MMP-9 - contribute to EMT activation (30, 47). MMPs regulate a variety of biological processes, especially those related to immunity and tissue repair and remodeling (35, 48). MMPs produced by myofibroblasts transdifferenciated from RPE could damage retinal tissue structure and induced fibrosis in nAMD.

TGFβ has three isoforms: TGFβ1, TGFβ2, and TGFβ3. TGF-β2 is more highly expressed than the other TGF-β isoforms, and its expression is correlated with retinal fibrosis (49). TGF-β2 plays an important role in the expression of downstream genes (50). TGF-β binds to its specific receptors and transmits signals downstream, causing nuclear translocation of the transcription factor Smad2/3 and leading to increased expression of various target genes that induce myofibroblast differentiation and production and secretion of extracellular matrix such as collagen and fibronectin, MMP production in RPE (35, 50). Our studies have also shown that TGFβ2-induced EMT in RPE cells involves the non-SMAD pathway, including mitogen-activated protein kinase pathway (ERK, p38, JNK) and AKT pathway, and can be attenuated by activation of the retinoic acid receptor-γ (RAR-γ) pathway (35). Moreover, the Jagged-1/Notch signaling pathway inhibits TGF-β2-induced EMT in human RPE cells (51). EMT of RPE during the progression of subretinal fibrosis in nAMD involves activation of SMAD-dependent and SMAD-independent pathways *via* TGF β. Moreover, TGF-βhave a profibrotic action, but it may not reflect the direct action of at least TGF itself. In skin, coadministration of CCN2 and TGF-β to subcutaneous tissue is necessary to achieve sustained fibrosis. Consequently, a role for CCN2 in enhancing TGF-β-induced fibrosis was proposed (52). In other words, EMT of RPE may be mediated through the secretion and action of other fibrotic effectors in addition to TGF-β.

TGFβ and the complement system are known to form cross-talk. In pulmonary fibrosis, immune complexes cause activation of complement, leading to fibrosis *via* a common signaling pathway with TGFβ1 (53). *In vitro* experiments suggest a positive feedback loop between TGF-β-mediated EMT and C5 production in RPE cells (54). Chronic inflammation is involved in the formation of subretinal fibrosis, and the complement system is implicated in chronic inflammation in many diseases, including AMD (55, 56). In a laser-induced mouse model of subretinal fibrosis, in addition to increased expression of TGF-β, VEGF, and fibroblast growth factor 2 (FGF2), which contribute to the process of fibrosis formation, expression levels of complement system factor C3 and complement factor B (CFB) are also increased (38). Dysregulation of the complement system has been identified as an important factor in inflammatory pathways in nAMD (57, 58). The concentrations of C3a, C4a, and C5a in peripheral plasma are significantly elevated in nAMD patients with subretinal fibrosis, suggesting that complement activation and the formation of anaphylatoxins such as C3a, C4a, and C5a may contribute to the development of subretinal fibrosis in nAMD, but the detailed mechanisms remain unknown (56). The C5a-C5aR pathway also induces EMT in RPE and is involved in the development of subretinal fibrosis (54).

Macrophages infiltrate the perifibrotic area in the retina and macrophages infiltrating retinal lesions are involved in fibrosis. Indeed, TGF-β and C3a, a complement system factor, can convert macrophages into myofibroblasts, which contribute to fibrosis (59). In contrast, in *in vivo* animal models of laser-induced CNV and subretinal fibrosis, administration of C3a blocking agent inhibited CNV formation but did not suppress fibrosis (38).

### TGF-β-mediated transcriptional regulation in EMT of RPE

**Figure 2:**
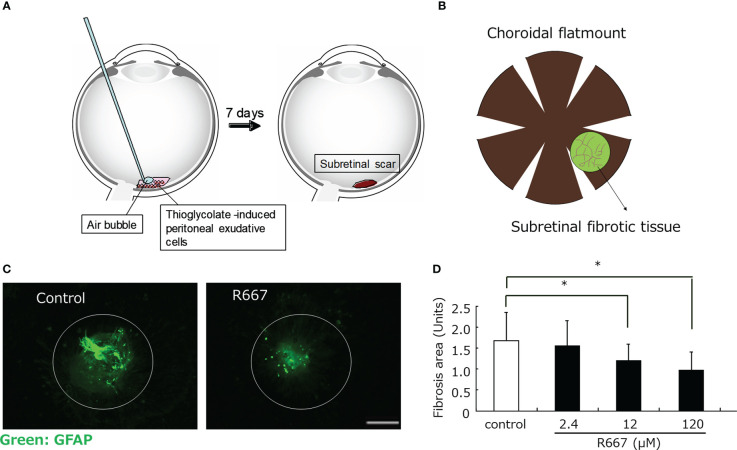
Inhibition of subretinal fibrosis by RAR-γ agonist(R667) in a mouse model. **(A)** Retina was subjected to laser photocoagulation (light wavelength, 532 nm; time, 0.1s; spot size, 75 μm; power, 200 mW) in order to generate a subretinal bubble and rupture Bruch’s membrane. Thioglycolate-induced PECs (4×10^7^/ml) were exposed to various concentrations of R667 in PBS, and 0.5μl of the cells was then injected into the subretinal space of the mouse recipients. Seven days after PEC injection, the animals were killed and the eyes were enucleated and fixed **(B)** Choroidal flat-mount preparations were washed with PBS, exposed to 100% methanol at room temperature for 10 min, and incubated first for 1 h at room temperature with 5% skim milk in PBS and then for 24 h at 4°C with **(C)** Choroidal flat-mount preparations from mice at 7 days after photocoagulation and injection of PECs treated with or without R667 (120 μM) into the subretinal space were subjected to immunofluorescence staining with antibodies to GFAP. GFAP was used as an indirect marker to better visualize fibrotic areas. Scale bar, 200 μm. **(D)** The area of subretinal fibrosis in preparations similar to those in **(C)** was determined for R667 concentrations of 0, 2.4, 12, or 120 μM. Data are means ± SEM for 10 eyes per condition. *P<0.05 (Dunnett’s test).

TGF-β activates the Smad signaling proteins, allowing them to translocate to the nucleus and participate in the transcriptional control of TGF-β target genes (60). In addition, TGF-β also activates SMAD-independent pathways, which regulate gene expression by activating specific downstream transcription factors. The TGF-β activation signaling pathway is associated with other signaling pathways and facilitates the induction of TGF-β-responsive genes through crosstalk between various transcription factors. Smad complexes translocated into the nucleus activated by TGF-β can regulate the activation of target genes by the interacting cofactors. Recently, transcription factors that promote EMT, such as Snail, Zeb1, Zeb2, AP-1, Sp1, and LEF1/TCF, have been found to interact as cofactors for Smads (61). TGF-β2 increases the expression of the transcription factors Twist, Snail, slug, and Zeb1 in RPE, followed by EMT (51, 62). The retinoic acid signaling pathway mediated by the retinoic acid receptor (RAR), which is a nuclear receptor, also affects the TGF-β/Smad signaling pathway through binding of RAR to Smad (63). We investigated the effect of parovalotene, a novel RAR-γ agonist, on TGF-β2-induced EMT of RPE by regulating Smad2 phosphorylation (**Figure 2**) (35). Although less potent than the RAR-γ agonist parovalotene, the RAR-α agonist AM580 also inhibited EMT of RPE induced by TGF-β2 and suppressed fibrosis in a laser-induced subretinal fibrosis model (37). In contrast, the effects of these agonists on CNV formation in laser-induced CNV models were examined; however, no inhibitory effect was observed. Recently, it has been reported that RAR-mediated retinoic acid signaling suppresses the TGF-beta/Smad signaling pathway and inhibits fibrosis in various cells (64, 65). One nuclear receptor, peroxisome proliferator activated receptor γ (PPARγ), forms a heteroreceptor with the retinoid X receptor (RXR) and is involved in gene expression. Furthermore, antagonists of PPARγ suppress EMT of RPE (66). During fibrosis, excessive secretion and accumulation of the extracellular matrix leads to structural changes and a more rigid structure. These mechanical actions by the extracellular matrix are transmitted through integrin receptors, which are cell adhesion structures and activate the YAP-Hippo signaling pathway, the SRF-MRTF signaling pathway, and its major downstream transcription factor groups, such as YAP, TAZ, SRF, and MRTF (67). They are activated and migrate to the nucleus, contributing to increased expression of fibrosis-related factors such as CTGF, which promotes myofibroblast proliferation and further activation (67). We found that TGF-β induces MRTF-A-dependent expression of EMT markers in human RPE. Furthermore, MRTF inhibitors significantly suppressed fibrosis in a laser-induced subretinal fibrosis model. Interestingly, MRTF-A inhibitors also did not affect CNV formation, suggesting that MRTF-A may also directly target subretinal fibrosis. Furthermore, YAP regulates the expression of EMT markers in RPE and is important for the TGF-β2-induced nuclear translocation and phosphorylation of Smad2/3 by TGF-β2 (68). YAP regulates EMT of RPE and plays an important role in tissue fibrosis. Therefore, YAP inhibition may be a target for suppression of subretinal fibrosis.

## Conclusion

In patients with nAMD, subretinal fibrosis is the most important factor determining final visual acuity (69). Anti-VEGF therapy, which is currently the first line of treatment, leads to visual improvement; however, the subretinal fibrosis that develops after treatment remains a clinical problem (70). Anti-VEGF therapy, when appropriately administered early in nAMD, plays a preventive effect against subretinal fibrosis by suppressing neovascularization (33). However, anti-VEGF therapy alone did not suppress subretinal fibrosis (71). Therefore, novel agents that inhibit subretinal fibrosis must be developed.

Myofibroblasts are mainly involved in the formation of subretinal fibrosis in nAMD and inhibition of myofibroblast differentiation, while activation is the key to treatment. Furthermore, myofibroblast-inducing factors (such as TGF-β, CTGF, PDGF, EGF, and FGF) and inflammatory factors (such as IL-6, S1P, and complement-related factors) have been reported. Representative molecular pathways involved in myofibroblast differentiation and activation include the Smad or non-Smad pathway initiated by TGF-β and Wnt/β-catenin signaling. Various antifibrotic compounds have been investigated as therapeutic agents that target these molecular pathways to inhibit retinal fibrosis in nAMD: TGF-β antagonists (68), PDGF-receptor-β antagonists (72), FGF2 antagonists (RBM- 007) (73), CTGF antagonists (74), interleukin-6 antagonists (75), and S1P antagonists (76).

**Figure 3:**
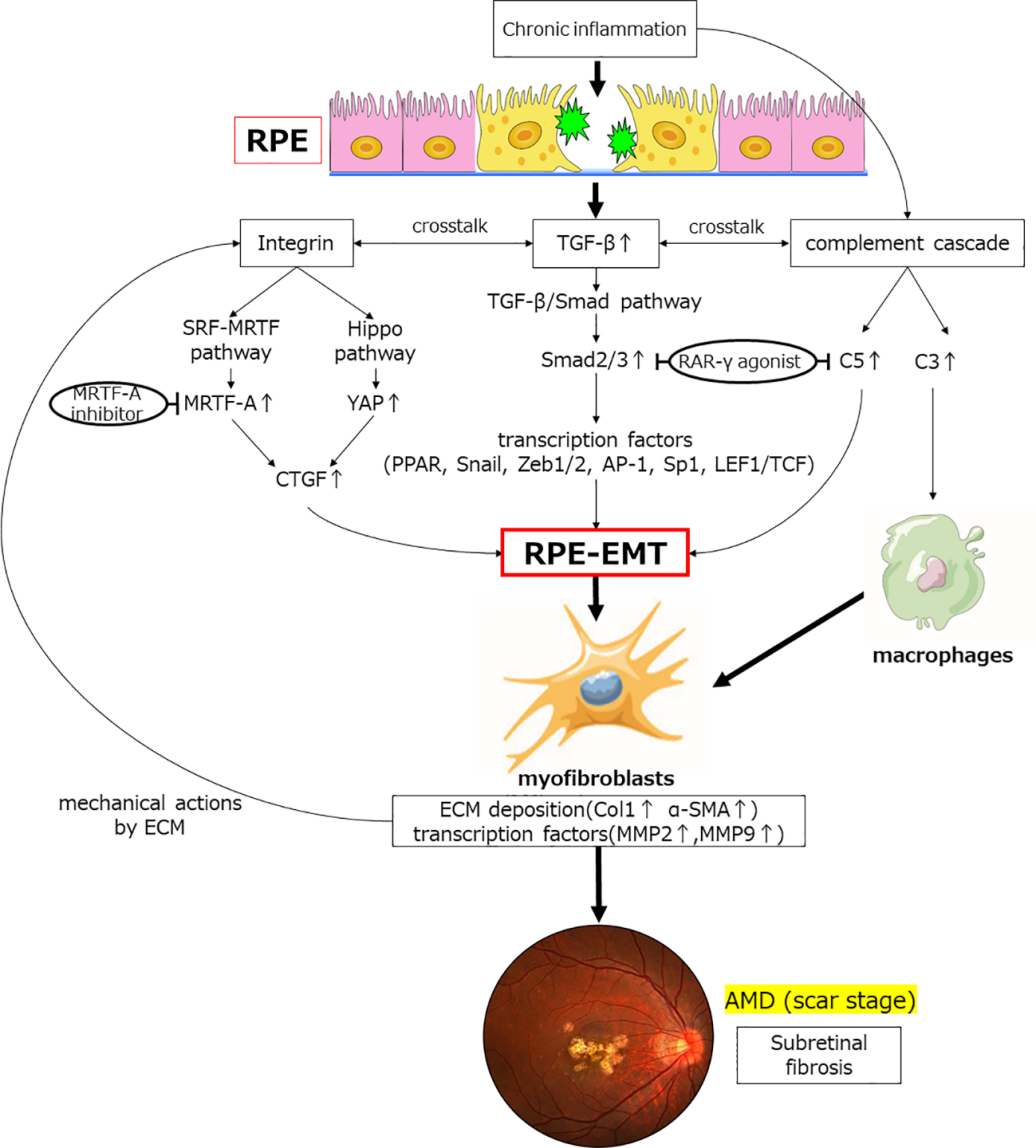
Inflammatory and transcription factors involved in TGFβ-mediated induction of EMT and subretinal fibrosis. Chronic inflammation increases the expression of the humoral factor TFG-β. Smad2 expression is upregulated *via* the TGF-β/Smad pathway, which induces EMT in RPE through expression of various transcription factors. The retinoic acid signaling pathway affects this pathway by binding of Smad to the retinoic acid receptor; TGF-β induces complement system factors in RPE. Furthermore, chronic inflammation also upregulates the expression of complement factors. C5 contributes to EMT in RPE, and C3 contribute to fibrosis by transforming macrophages into myofibroblasts. Crosstalk between TGF-β and integrins activates the Hippo signaling pathway, the SRF-MRTF signaling pathway, and its major downstream factors (YAP/TAZ, MRTF) during fibrosis. These are activated and translocated into the nucleus, contributing to elevated expression of factors associated with fibrosis, such as CTGF, which affect EMT in RPE. Myofibroblasts so generated result in deposition of excess ECM and elevated expression of transcription factors. Physical stimulation with excess ECM further induces integrin-mediated EMT of the RPE. Finally, subretinal fibrosis of AMD is formed. RAR-γ agonist and MRTF-A inhibitor are potential therapeutic agents that may inhibit EMT in RPE.TGF-β, transforming growth factor-β; YAP, Yes-associated protein; MRTF, myocardin-associated transcription factor; CTGF, Connective tissue growth factor; RAR, retinoic acid receptor; PPAR, peroxisome proliferator activated receptor; EMT, epithelial-mesenchymal transformation; MMP, matrix metalloproteinase; Col1, collagen type 1; α-SMA, α-smooth muscle actin.

In addition to transcription factors (such as Snail, Zeb1, Zeb2, AP-1, Sp1, and LEF1/TCF) that act as coactivators of Smad, a nuclear receptor, PPAR is an important therapeutic target in RPE and its expression is induced by TGF-β to differentiate and activate myofibroblasts in RPE. It is involved in the differentiation and activation of myofibroblasts in the RPE. RAR-γ agonists suppress EMT in RPE cells and significantly inhibit the formation of subretinal fibers in an *in vivo* subretinal fibrosis model. RAR-γ agonists significantly inhibit TGF-β-induced complement C5 expression in RPE cells. This indicates that RAR-γ agonists may be involved in the complement-mediated macrophage-derived myofibroblast pathway, including C5 and Smad2-mediated RPE-derived myofiber formation. The MRTF signaling pathway is also activated by TGF-β, which induces EMT in RPE and suppresses subretinal fibrosis, suggesting that MRTF-A inhibitors may also be important targets in the future. This suggests that MRTF-A inhibitors may also be an important target in the future (36). Furthermore, the YAP-Hippo signaling pathway may be a novel target for future studies on the inhibition of subretinal fibrosis, as MRTF is also a transcription factor involved in mechano-stress (**Figure 3**).

Numerous aspects of fibrosis formation remain unknown; for example, why does the inflammatory response triggered by fibrosis does not converge while chronic inflammation persists, and how does tissue remodeling occur and ultimately lead to tissue dysfunction. It is believed that new findings will help researchers to continue to elucidate the fibrosis mechanism at the molecular level, thus leading to the development of novel methods for the prevention and treatment of subretinal fibrosis.

TGF-β plays a central role in the EMT-induced transformation of RPE cells into myofibroblasts, as well as in subretinal fibrosis formation. Studies of fibrotic diseases in other tissues and organs have also shown that the TGF -β signaling pathway is closely related to fibrosis; TGF-β is involved in the regulation of multiple intracellular signaling pathways, not only in EMT and fibrosis, but also in wound healing, metabolism, aging, and epigenetics, and is important in maintaining homeostasis It also plays an important role in maintaining homeostasis. Indeed, some therapeutic agents that block the TGF-β signaling pathway, such as pirfenidone, have effective antifibrotic properties, but side effects are a major problem (77). Therefore, finding targeted signaling pathways to treat fibrosis while avoiding toxic side effects is important in drug development. Therefore, combination therapy may be one option in the treatment of subretinal fibrosis. nAMD produces subretinal fibrosis secondary to CNV, so a combination of CNV-suppressing VEGF and inhibitors targeting the TGF-β signaling pathway may be an option. On the other hand, the timing of administration of these agents should be carefully considered, as the time of disease progression of nAMD differs somewhat from the time of the pathological condition in which they act. Regarding combination therapy, a rational combination of other drugs with different mechanisms of action from the TGF-β signaling pathway, along with inhibitors that target the TGF-β signaling pathway, may be necessary in the treatment of subretinal fibrosis in the future. It will be important to continue to ascertain whether drugs that inhibit TGF signaling or related signals can be used therapeutically or prophylactically for subretinal fibrosis associated with wAMD. During the formation of subretinal fibrosis in nAMD, there are still many unknowns, such as why the inflammatory response triggered by fibrosis does not converge and chronic inflammation persists, and how tissue remodeling occurs, ultimately leading to tissue dysfunction. It is hoped that new findings will continue to elucidate the mechanism of fibrosis at the molecular level and lead to new methods of prevention and treatment of subretinal fibrosis.

## Author contributions

KK designed whole the study. FH and MH, TY, YK, MW drafted the manuscript. KK reviewed the studies and performed the systematic review. All authors contributed to the article and approved the submitted version.
